# The Mystery at Silver Cliff

**Published:** 1876-08

**Authors:** Madge Nasheé


### *THE MYSTERY AT SILVER CLIFF.*

By Madge Nasheé.

went away.

"I was thoroughly ashamed of myself the next morning, and vexed to see Mark looking pale and distrait. I had worried him with my nonsense unnecessarily, I thought.

"Well, several days afterward, Laura told me the story of Sir Piers Leigh, and why his spirit was supposed to be unquiet. There were records of his appearing to various members of the family at long intervals since his own violent death, and his presence in visible shape always portended an uncanny end to the person so visited.

"Mark laughed at it all, and declared that there was nothing in it; but Mrs. Leigh looked very grave, and the subject was dropped.

"Perhaps I was a foolish, fanciful girl. I dare say I was; but I could not get over my terror of the ghost, nor the thought of the Indian Mandomin. It seemed to me that there was something more in their manner to him than dread of what a stranger might do, and I dared not tell Mark what I felt. If ever I mentioned his name, a hard expression came into my husband's face and a troubled look into his eyes that silenced me at once.

"Mark had some engagement that would take him away from me for about six months. After that he would do nothing that would separate us. So it was arranged that he should take me to Montreal, and find me a home there with some nice people whom he knew, who would prevent my being dull, and where he could see me once at least during the time.

"I left 'Silver Cliff' without having heard any more of the secret of which they had spoken on the night of my arrival, and which I naturally concluded to be the story of the family ghost. I forgot it in the joy of getting back to the civilized world again, and was as brightly happy as on my wedding morning.

"I saw Mark once. He came to me for two whole days, full of glowing prospects for the future so near now when we should be always together. It was the *last* time before — —"

She shuddered and hid her face. "Never mind it, dear child," I said, my voice full of the sympathy I felt for her. "Don't tell any more if it makes you feel so badly."

"No, no, it does me good to tell you," she said, drawing closer to me; "only what I have to say is strange and awful. If it were not that I was all alone, that I stood by and saw them close his coffin-lid, and then put him into his grave, I should think it all some foul, hideous dream, from which I should awake in his arms.

"I say I never saw my husband again. I know now that I did not, until I saw him in the one awful moment when time and eternity join, and this life is laid down forever; but knowing what I know, with the terrible secret of the past revealed to me, I can hardly believe that I did not see him one winter night in Montreal only a short week before he was to have come back to me to go away no more.

"Winter was setting in, for the six months had grown into ten; Mark's engagement being lucrative, but it was over now, and he was coming back to me. I was alone, but not lonely. I had found friends in the family in whose house I lived. I had been sleighing with them, and even then, though it was night, the jingle of sleighbells could be heard all through the street, telling of moonlight parties and pleasant meetings.

"I was sitting without a light—I always had a *penchant* for moonlight—watching the sparkles on the edges of the ice-blocks in the river, and lost in a queer reverie of the spring, when the ice bursts so tumultuously, and sets the imprisoned water free. A slight stir in the room aroused me, and turning my head I saw Mark. With a glad cry, I sprang to him and clasped him in my arms. He did not repulse me, but he did not return my caress, and there was something strangely wild in his looks.

"'My darling—my darling!' I said, 'what a joyful surprise! How good of you to come!'

"'It's only for a few minutes, Daisy,' he replied, hoarsely. 'I must be off again. I want whatever money you have handy.'

"I had a large sum in my desk. Mark had supplied me liberally, and left me blank checks as well.

"'Here is what you gave me,' I said; 'nearly all of it.'

"Something in his wild, disordered appearance made me feel sure that he had been drinking. I was terribly frightened, and my former employer's words rushed into my mind.

"'Mark, dear, what has happened?' I asked, laying my hand on his arm. He put me away, not roughly, but coldly, with a hand that burnt like fire, and shook as though with the ague.

"'There's nothing the matter,' he replied.' 'Give me the money quick. I must go. I have people waiting for it and me.'

"His tone was so curt and unkind that my eyes filled with tears. 'What makes you so harsh to me, Mark?' I asked.

"'I'm not harsh, child. There, don't be a little fool,' and he stooped toward me and kissed me lightly on the forehead. 'Don't worry your little head about me, but look for me when I said I would come. By-by!'

"I did not hear him go out, for I was crying as I think I never cried in my life before, over his strange and altered manner. I was better next day and tried to reason myself out of my fancies, and to anticipate his coming with the same joy I felt before. But he never came—no sign, no letter; and I was nearly frantic with grief and terror. I did not know where to send, nor could they tell me at the office where his employers heard from him. He had disappeared in a most unaccountable manner. Sir Piers Leigh told me where to find him."

"The *ghost*, Daisy?" I ventured to remark.

"Yes," she answered, "the ghost, if it was one—I don't know. If it was fancy, it was a curious one, and a strange coincidence."

She spoke dreamily and absently, as though she was far away, and as if she could see the spectre of Piers Leigh in actual shape before her.

"You see, I had been alone so long, and I was getting so nervous and frightened at Mark's being away, and not knowing where to find him, that I don't think I was quite myself. I wrote to my mother-in-law at Silver Cliff, but she did not answer, and the people of the house advised me to go to her. I suppose I ought to have done so; but my dread of that house was growing greater instead of less as the time went on. But I felt that I must go somewhere or do something; and at last I told them that I would go if another week passed away, and I heard nothing from Mark.

"I think I was mad that night. I remember pacing up and down my room in the moonlight, sobbing, and calling wildly on my husband.

"'Oh! Mark, my darling!' I called, 'where are you?'

"And a voice that I rather felt than heard, though it was distinct and clear, said:

"'At Hamilton!'

"I stopped in my wild walk, not frightened, but stricken dumb, for Piers Leigh stood between me and the light, exactly as I had seen him at Silver Cliff, when he had come and laid his hand on Mark's shoulder.

"Another moment and he was gone, and the moonbeams were flooding all the room as before, and the name of Hamilton was ringing in my ears. I knew there was such a place somewhere on Lake Ontario, and that was all I knew about it. And so the week passed over and every night I dreamed of Mark. He was always calling me, and I was always trying to ask him where he was, but could never frame the words. I was to start for Silver Cliff on the morrow, to be there for Christmas Day, I had quite made up my mind, and again I dreamed of Mark, pale and imploring.

"'Come, Daisy,' he said; 'come at once, or it will be too late!'

"'Where—oh, where?' I cried, bursting the strange bonds that seemed to hold my speech; and he answered:

"'Hamilton!'

"It was the same curious voice I had heard before, and I woke with a gasp and a shudder, to see the shadow of his ancestor standing by my side.

"'Go to Hamilton,' he said, distinctly. 'He is there.'

"I did not tell the people of the house of my changed plan, but I went on the road the mysterious warning pointed—a long, weary journey—two days' incessant travelling, flying past towns and cities, full, doubtless, of busy, eager people, but with no tittle of interest for me. I don't remember much about the time, save that it lagged most horribly. The trains seemed to creep, and the country, when the towns became fewer, seemed a desolate, snow-engulfed wilderness. People got in and out, and talked, and the farther we went the oftener I heard a name repeated, which seemed to have some curious interest for every one—'Lance Linwood.' I was too stupid to understand at first, but I gathered by and by that the name belonged to a most atrocious criminal, who was in prison, somewhere, awaiting his deserts.

"I remember going to Hamilton, and hearing people say it was a pretty place, and seeing people stare at me curiously, seeing me unattended, and so forlorn-looking. I had plenty of money. Mark had taken care of that, Heaven bless him, and my wits were steady enough still to enable me to take care of myself. There were sleighs in plenty at the station, and I got into one, and bade the man drive me to the best hotel in the place. I had sense enough left to know that rest and refreshment must come even before my search for my darling. I remember being driven along a fine esplanade, with a handsome fountain, all frozen up now, upon it. The place seemed very full of people, and I remarked upon it to the driver.

"'They've come to see the hanging,' he said. 'Lance Linwood was to be hung to-day. It's all over by now, ma'am. It was to be at eight o'clock, and the world's well rid of him, I say.'

"I had gathered from the talk coming along that this Linwood was a most atrocious criminal, and that the particular offence which was to cost him his life was a murder of almost unparalleled barbarity. Our way lay past the place of execution, but the driver declared the crowd would have in a great measure dispersed by this time. But it was impassably dense, and right opposite the jail we were completely blocked in.

"'I made a mistake, miss,' the man said. 'It isn't over. I'm very sorry.'

"He didn't look so, and I've thought since that he went that way on purpose that he might see what was going on. He offered to take me into a house till I could get on; but I was afraid to press through the mob, who were in a state of the wildest excitement.

"It was an awful thing to think of a man being hung at such a time—on the very eve of the day of 'peace and good will' to all on earth; and I remember thinking so in a dazed sort of way, and wondering whether the poor wretch had any one to grieve for him. But I was getting stupid with my own grief, and Lance Linwood was nothing to me.

"A yell of execration, that seemed to shake the very earth, burst from them as a bell began to toll, and one or two people appeared on the little scaffold above our heads. I looked up, fascinated, as every one is fascinated by horrors, at the man who was to die, on whose face the winter sunshine was shedding a halo of golden rays.

"Great heaven! It was Mark—my husband—about whose neck the hangman's hands were busy with the loathsome rope. It was my darling who stood there with pinioned arms, looking his last upon the world of light and love, and leaving me behind in the cold and darkness.

"Again the people yelled as if though their throats would crack, but my scream, my agonized cry, 'Oh, Mark! Mark!' rose above it, and reached him. He heard it, for his dear face brightened for a moment, and then grew drawn and dark with agony. His body swayed, as though he were trying to stretch out his arms to me, and then I saw the white cap in the hangman's hand, and knew no more.

"When I came to myself, I was lying on a sofa, in a neatly-furnished parlor, looking into a gloomy court. The windows were barred, but the room itself was cheery and homelike, and a woman was in it—a pleasant, motherly woman, with tears in her kindly eyes—doing all in her power to aid and rouse me.

"'Poor thing,' I heard her say, before I could move my numbed limbs. 'She's a delicate, ladylike creature. There must be some awful blunder somewhere—there must, James.'

"'There isn't,' a man's voice said. 'This poor lady is Lance Linwood's widow, there can be no doubt. All the country knows now that the arch villain led two lives. At home his name was Leigh, and he was a good son and a respectable member of society; abroad—well, the murder of the Darches, and the burning of their settlement, show pretty well what he was. The world is well rid of him.'

"And I heard this, and was powerless to speak and shriek out that it was a lie. I was a widow, then, and Mark, my Mark, was waiting the dog's burial that the law accords to those whom it kills. I had been brought into the prison by a gentleman who had heard me shriek out that they were murdering my husband, and who had seen the awful look of recognition on Mark's face, as he heard my cry. The woman who was ministering to me was the sheriff's wife, and her husband had just come in from his dreadful duty, and was warming himself at the fire. I think I should have died where I lay, unable to move or speak, but that the sound of scuffling and screams became audible in the passage—shrieks in a voice that I knew well—the voice of my sister-in-law, Laura Leigh.

"'I will see the sheriff!' I heard her say. 'I will stand face to face with the man who has murdered my brother.'

"And, not knowing that I was there, the turnkey knocked at the door and admitted her. It was Laura, in very deed, soiled and travel-worn, with an unnatural gleam in her dark eyes, and great streaks of white in her black hair. She seemed almost beside herself, and evinced no surprise at seeing me.

"'She here?' she said. 'Poor girl! poor girl! another innocent one to suffer for the guilty. Am I in time? They said out yonder that the foul thing was done, and that you had killed my brother. It is not true, of course, or you would not stand there looking so coldly and placidly at me as you do.'

"'Poor soul!' said the sheriff's wife. 'Sit down and don't take on so. It is all over, dearie, and may be it's best. No good ever comes of a dreadful parting when there's no hope on either side. He didn't suffer. It was all over in a minute.'

"She put Laura into a chair, for she looked quite stupified, and the sheriff went to her kindly enough

"'It is over, my dear lady,' he said; 'Lance Linwood is dead.'

"'Lance Linwood!' she repeated, with a bitter smile. 'Aye, there it is! You think you have killed the man who murders people in their beds, and burns their homes about them for the sake of gain. You think you have done the State good service in ridding the world of the monster to whom the life of a helpless infant was not a sacred thing, who would take a babe out of its mother's arms, and dash its head to pieces against the wall if the fancy took him. I'm not mad, Daisy,' she added, suddenly turning to me, seeing my terrified stare. 'At least, not yet. They will try and persuade you that this incarnate fiend was your husband. Don't believe them, my girl. Lance Linwood is alive and well, and Mark—our Mark —has died in his place.'

"I could not speak. I thought I was in a dream or mad; but the sheriff looked very dark and stern, and his wife was as white as a sheet.

"'Try and talk calmly, my girl,' he said. 'This is a terrible statement to make.'

"'It is true,' she said. 'Lance Linwood has been in my mother's house a week. We did not know of what was happening here. How should we? We have been away in the States these three months. Four days ago the dreadful knowledge came to us, and I came here. You can stop him; there is time. He will leave New York in the Europa to-morrow, taking the name of this poor child's dead husband, Mark Leigh.'

"'I can not understand,' the sheriff said, in a low tone.

"'I think I can,' said his wife, quietly.

"'But did not your mother, knowing who he was, and what he had done, hand over this felon?' he asked, somewhat sternly. 'Knowing, too, that another man was suffering in his place.'

"'We did not know—we knew nothing until the appalling tale came to us four days since. My mother dropped where she sat, stricken with her death blow. She will never speak again—perhaps she will die all alone at Silver Cliff while I am here, who knows?' Laura went on, in a voice whose wrung agony told of what she was enduring.

"'But why did you let him stay in your house, young lady?' What motive could your mother have for giving shelter to a man whose life has been forfeited to offended justice any time these half-dozen years?"

"'What motive?' and Laura's slight form reared itself as if in indignation at the question. 'What motive has the wild beast of the field for shielding and sheltering its young? He was her son, too —outcast and criminal though he was, he was her pure and innocent baby once—twin brother of the man whose life has paid for his this dreadful day.'

"I started up with a wild scream. I knew all the whole terrible truth now. The secret that had been so carefully kept from me was the fact of the existence of this evil twin brother of Mark's. I learned afterward what a thing of guile and wickedness he had been from his very cradle. It was as if a devil had been born into the peaceful family which nothing could exorcise. I knew the mystery of the odd picture at Silver Cliff now; the angry father had had the face of the boy whose misdeeds helped to bring him to his grave effaced from the pure companionship of his brother and sister; I knew, too, who the Indian was who had levied blackmail from those helpless women, and why my husband was credited with roistering and dissipation that he had no part in.

"I heard all about the arrest and false accusation of my innocent husband when I was able to hear it. A series of accidents which no one could foresee or avert had prevented a letter from him reaching me for some time after it happened, and when it did he would not alarm me with the story.

"He should be free directly, he said; but circumstantial evidence arose in overwhelming mass, and he could not prove his innocence. He was alone on the night of the murder, camping out with no company but his dog, and a score of witnesses swore to his identity with Lance Linwood. One of the victims of the outrage identified him with his dying breath, and two men who had been concerned with him in more than one piece of villainy declared he was their comrade. They were so alike these twin brothers! Justice is sharp and speedy in these Western cities, and only a fortnight elapsed from the time of his arrest to the day when I saw him in the hangman's hands on that bright winter morning. He would not have me told, he said; it was better I should think him false—dead—guilty of any sin toward me than know that he died a dog's death on the public scaffold.

"They were very kind to me, the people at the prison, they gave Laura and me our dead, to bury out of sight. Kindly hands had straightened Mark's limbs, and laid him in his coffin before we looked upon his beloved face, and the tragedy of the morning had left no traces but such as could be hidden by the last garments of poor humanity. He must have died instantly.

"If kindness and sympathy could have atoned to me for the wrong done me in the name of justice, or if money could have repaid me for my Mark's loss, I should be a rich woman now; but all I wanted when I turned away from his grave was home, and to weep out my grief in my auntie's arms. I heard in New York that Lance Linwood had been caught, and so nearly torn to pieces by a furious mob before the police could rescue him that he died as they were carrying him into the prison. I didn't see Laura any more; they took her back to Silver Cliff, poor thing; but Mrs. Leigh is dead. She never knew that her other son had met with his deserts."

When Daisy finished her sad story silence fell upon us for a long time. What comfort could my sympathy be to one so *cruelly* bereaved. A knock at the door startled us, and opening it I found Mrs. Blake, who, having become anxious at the prolonged absence of her niece, had come in search of her. Daisy arose, I silently kissed her, and she departed with her aunt, leaving me alone with my gloomy thoughts.

---

### *TO MY WIFE ON HER BIRTHDAY.*

By W. Waybridge, Esq.

The song birds come and go,
The rose, the winter snow;
  But still the same,
My heart, dear wife, for thee
Beats fond and lovingly
As when first given to me
  To speak that name.

Ere life had well begun
Our souls were blent in one
  Sweet song of love;
And as time sped away,
How bright the golden ray
That o'er us day by day
  Shone from above.

If tears were sometimes sent,
As when our darling went
  To his long home;
The grief rose not in vain,
Diviner is the strain
Of loved ones that remain
  O'er earth to roam.

Now seas between us roar,
But on this distant shore
  I think of thee
As a bright star that Heaven
Has to a wanderer given
To soothe his spirit riven.
  Oh, think of me!

Italy, May 6th, 1875.

